# A social-architecture perspective on gut microbiota dynamics and host physiology

**DOI:** 10.3389/fimmu.2025.1642080

**Published:** 2025-11-17

**Authors:** Zhexin Ni, Wei Zhou, Yue Gao

**Affiliations:** Beijing Institute of Radiation Medicine, Beijing, China

**Keywords:** gut microbiota, microbial society, cooperative networks, competitive exclusion, host health

## Abstract

The human gut microbiota, a dynamic consortium of trillions of microorganisms, is increasingly recognized not merely as a metabolic entity but as a structured “microbial society” exhibiting hierarchical organization, cooperative networks, and competitive exclusion. This hypothesis posits that gut microbiota communities operate under principles analogous to social structures, with emergent behaviors that directly impact host health. By integrating recent advances in microbial ecology, spatial omics, and neurogastroenterology, this paper proposes those microbial social dynamics—such as division of labor, territorial specialization, and collective decision-making—mediate critical host functions, including immune regulation, metabolic homeostasis, and cognitive processes. In research or therapy targeting the gut microbiota, safeguard the stability of the microbial society and eschew simplistic, blunt approaches. In short, the gut microbiota behaves like a collective mind, showing tight unity and rapid, fine-tuned adaptation to external cues. Its imbalance breeds disease; its vigor enhances human life.

## 1 Introduction

**Figure 1:**
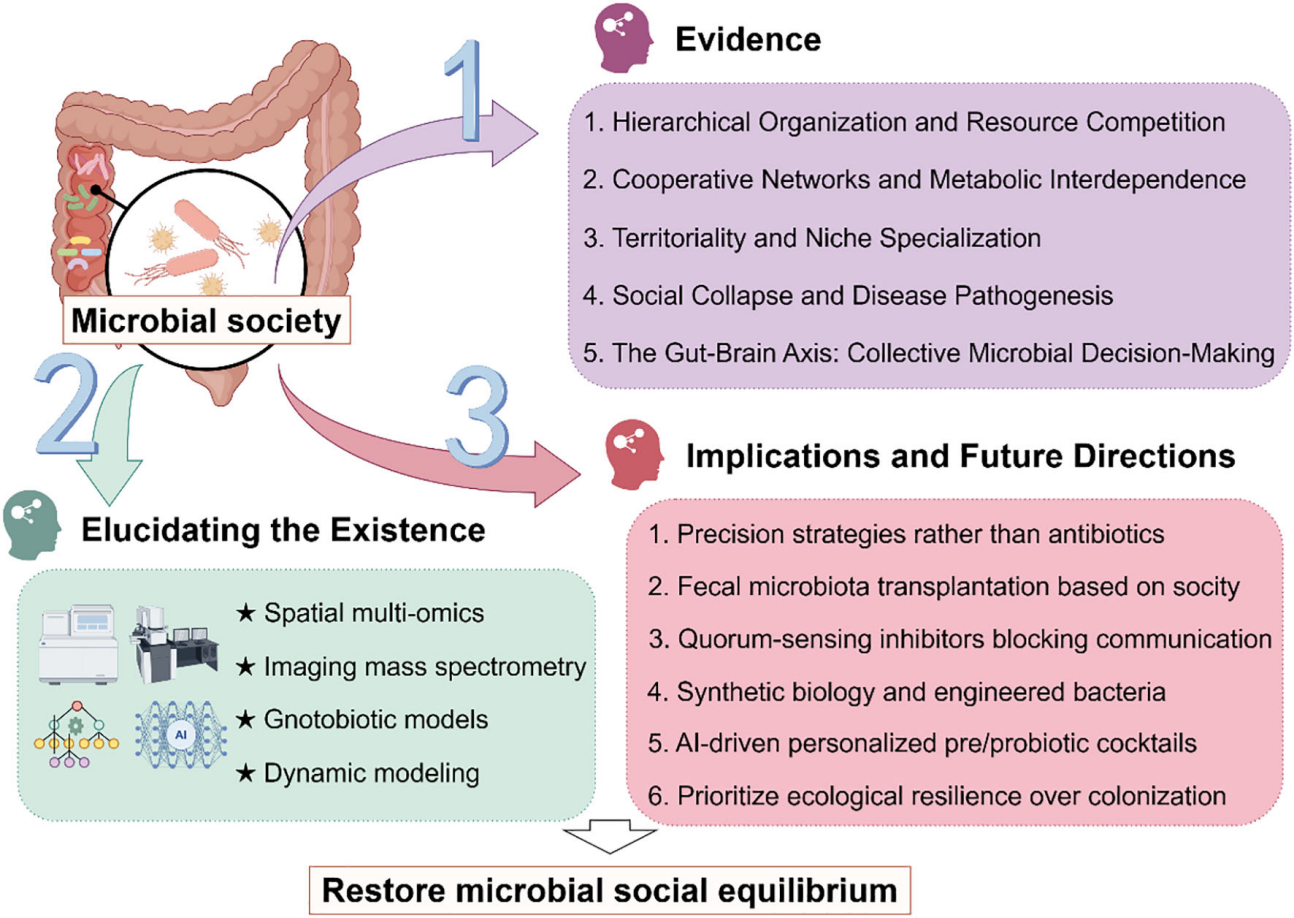
Research framework for investigating social dynamics in gut microbiota. Gut microbiota constitutes a vast, complex ecosystem with evidence suggesting intrinsic social organization. While current observational methods are insufficient to fully decipher their social dynamics, emerging approaches such as spatial multi-omics and dynamic modeling can gradually elucidate their mechanisms. These insights will inform the development of ecological modulation strategies to stabilize microbial communities, ultimately supporting human health.

The human gut microbiota comprises complex microbial communities that interact dynamically to form intricate ecological networks (1). Emerging evidence suggests that these microbial populations do not exist as random assemblages but may instead self-organize into structured social architectures characterized by hierarchical dominance, mutualistic alliances, and niche-driven territoriality (2). Such organization is critical for maintaining gut homeostasis, with disruptions to these social structures potentially contributing to dysbiosis-associated diseases, including inflammatory bowel disease (3), metabolic disorders (4), and neurological disorders (5). The stability of these microbial social frameworks is maintained through sophisticated communication mechanisms, such as quorum sensing and metabolite exchange (6), which facilitate cooperation, competition, and spatial organization. Furthermore, host-related factors—including anatomical constraints (7), dietary inputs (8), and immune-mediated selection pressures (9)—play a pivotal role in shaping these microbial interactions. Despite growing recognition of the gut microbiota’s social dynamics, the precise rules governing their organization and how their breakdown leads to disease remain incompletely understood (10). Therefore, we propose the hypothesis that gut microbiota communities exhibit socially structured behaviors analogous to macro-ecological systems and that disturbances in these organizational principles underlie pathological dysbiosis. By elucidating these mechanisms, this perspective paper aims to provide novel insights into microbiome-based therapeutic strategies (**Figure 1**).

## 2 Evidence supporting microbial social structures

### 2.1 Hierarchical organization and resource competition

Dominant bacterial phyla (e.g., Bacteroidetes, Firmicutes) act as “keystone species,” shaping gut microbial community composition through sophisticated resource competition mechanisms—this hierarchical control is not merely numerical dominance but functionally orchestrated to regulate nutrient fluxes and niche occupancy. As the primary drivers of community structure, these phyla employ substrate monopolization, microenvironmental modulation, and spatial exclusion to maintain their ecological status, mirroring how core institutions govern resource distribution in human societies.

A quintessential example is *Bacteroides thetaiotaomicron*, a flagship species of Bacteroidetes, which dominates polysaccharide metabolism via an elaborate repertoire of 88 polysaccharide utilization loci (PULs)—genetic modules encoding carbohydrate-active enzymes, transporters, and regulatory proteins (11). This PUL system enables it to rapidly degrade both dietary polysaccharides and host-derived mucin glycans: when dietary fibers are abundant, it prioritizes degrading starch via the StcP PUL, achieving 90% substrate utilization within 4 hours in anaerobic batch cultures; when dietary resources are scarce, it switches to mucin degradation via the MucP PUL, sustaining metabolic activity while outcompeting nutrient-limited taxa (12). Additionally, *B. thetaiotaomicron* modulates the local microenvironment to reinforce dominance: its fermentation of polysaccharides produces short-chain fatty acids (SCFAs), lowering luminal pH to 6.5–7.0—a range that inhibits acid-sensitive competitors (13).

Spatial stratification further reinforces this hierarchical resource allocation, with microbial distribution tightly linked to resource accessibility and microenvironmental gradients. Oxygen-sensitive *Clostridia* (a Firmicutes class) occupy hypoxic deep crypts, where they utilize SCFAs produced by upper-layer taxa for butyrate synthesis; in contrast, facultative anaerobes dominate oxygen-rich luminal regions, prioritizing glucose scavenging via high-affinity glucose transporters (14). This stratification extends across intestinal segments: the small intestine exhibits a proximal-to-distal biomass gradient driven by declining gastric acid concentration and increasing nutrient availability, with proximal regions dominated by fast-growing nutrient specialists (e.g., *Lactobacillus rhamnosus*) and distal regions hosting diverse anaerobes (e.g., *Eubacterium rectale*) that compete for complex polysaccharides (15).

Keystone species also enforce hierarchy via antagonistic metabolic interactions. For instance, *B. thetaiotaomicron* expresses bile salt hydrolases that convert host-secreted primary bile acids (e.g., cholic acid) to secondary bile acids (e.g., deoxycholic acid) (16). These secondary bile acids inhibit opportunistic pathogens like *Clostridioides difficile* (by disrupting its cell membrane integrity) and suppress subdominant Firmicutes species (e.g., *Ruminococcus torques*) that lack bile acid resistance genes, further consolidating *B. thetaiotaomicron*’s dominant niche (17). Such multi-faceted resource competition ensures that keystone phyla maintain community order, with spatial and metabolic strategies collectively reinforcing a hierarchical structure where resource access directly dictates ecological status.

### 2.2 Cooperative networks and metabolic interdependence

Metabolic interdependence via cross-feeding relationships constitutes the core of microbial cooperative networks, where taxa exchange essential metabolites to fill functional gaps, stabilize community structure, and amplify beneficial effects on host physiology. This mutualism is not random but evolutionarily optimized, with each species contributing a unique metabolic function that complements its partners—mirroring specialized roles in human societies.

A paradigmatic example is the synergy between *Akkermansia muciniphila* and *Faecalibacterium prausnitzii*. *A. muciniphila*, a key mucin-degrading bacterium, expresses a suite of mucinases that hydrolyze host-derived mucin glycans into N-acetylglucosamine, fucose, and acetate (18). Transcriptomic analysis of co-cultures shows that acetate secreted by *A. muciniphila* upregulates the expression of butyrate synthesis genes in *F. prausnitzii*—specifically *butA* and *crotonase*—increasing butyrate production compared to *F. prausnitzii* monocultures (19). Functionally, this synergy directly benefits the host: butyrate enhances intestinal epithelial barrier integrity by upregulating tight junction proteins and reduces pro-inflammatory cytokine secretion in Caco-2 cell models (20). A human cohort study further confirmed that co-abundance of *A. muciniphila* and *F. prausnitzii* correlates with lower intestinal permeabilityand a reduced risk of irritable bowel syndrome (21).

Another well-characterized cooperative pair involves *Bifidobacterium longum* and *Eubacterium hallii*, which form a metabolic chain centered on dietary fiber. *B. longum* degrades complex fibers via β-fructosidase to produce lactate (22). *E. hallii* then utilizes this lactate through the lactate dehydrogenase pathway, converting it to propionate—a critical immunomodulatory metabolite (23). Mechanistically, propionate acts on host immune cells by inhibiting histone deacetylase 6 in macrophages, suppressing the production of TNF-α and IL-6, and promoting the differentiation of regulatory T cells (24). This immunoregulatory effect was validated in a randomized controlled trial, where supplementation with a *B. longum* reduced colonic inflammation in patients with active ulcerative colitis (25).

Beyond pairwise interactions, multi-species cooperative networks further enhance community resilience. For instance, *Ruminococcus bromii* releases maltose oligosaccharides that fuel *Bacteroides ovatus* (26), which in turn secretes succinate (27). This succinate is then utilized by *Methanobrevibacter smithii* for methane production (28), creating a three-species metabolic loop that improves overall fiber fermentation efficiency. Such multi-partner interactions highlight the complexity of microbial cooperation, where each species acts as a “metabolic node” to sustain the collective function of the microbial society.

### 2.3 Territoriality and niche specialization

The gut’s inherent anatomical heterogeneity—encompassing gradients in bile concentration, oxygen tension, nutrient availability, and epithelial structure—creates distinct microhabitats that foster microbial “tribes,” each adapted to specific ecological niches through phenotypic and metabolic specialization. This territorial partitioning is not random but evolutionarily optimized to minimize interspecies competition, maximize resource utilization efficiency, and reinforce community stability, analogous to how geographic and environmental barriers shape distinct human societies.

The ileum and colon, as functionally divergent intestinal segments, exemplify this niche specialization. The ileum, positioned at the junction of the small and large intestine, is characterized by high bile acid concentrations and a nutrient pool dominated by partially digested carbohydrates and dietary amino acids (29). These conditions select for bile-tolerant taxa like *Enterobacteriaceae*, which encode specialized adaptive mechanisms: bile salt hydrolases that deconjugate primary bile acids (e.g., cholic acid) into less toxic secondary forms, and AcrAB-TolC efflux pumps that expel excess bile acids from the cell (30). A human study confirmed that BSH genes are widespread among 591 gut bacterial strains across 117 genera, with the highest prevalence found in Bacteroides species (including bshA homologs), and that BSH gene activity varies across different intestinal regions (31). The colon—rich in undigested dietary fibers, hypoxic, and neutral pH—favors fiber-fermenting taxa like *Lachnospiraceae* (32). Genomic analysis of *Lachnospiraceae* strains reveals an expanded repertoire of carbohydrate-active enzymes (CAZymes), including endo-β-1,4-glucanases (for cellulose degradation) and α-L-arabinofuranosidases (for hemicellulose degradation), with an average of 40–50 CAZyme-encoding genes per genome. This specialization enables *Lachnospiraceae* to dominate the colonic microbiota and produce butyrate via fermentation, further reinforcing their niche by supporting epithelial health (33).

Advanced imaging techniques have resolved the spatial precision of this territoriality, particularly in microbial biofilms. Confocal Laser Scanning Microscopy combined with Fluorescence *In Situ* Hybridization has visualized spatially segregated biofilms in the colonic mucosal layer (34): consortia of *Bacteroides* (e.g., *B. thetaiotaomicron*) occupy the inner mucus layer (35), while *Prevotella* (e.g., *P. copri*) dominate the outer mucus layer (36). Metabolic profiling of these niche-specific populations reveals functional complementarity: *Bacteroides* express high levels of mucin-degrading enzymes (e.g., α-fucosidases, β-galactosidases) to utilize host-derived mucin glycans (37), while *Prevotella* prioritize dietary fiber degradation via xylanases and arabinases (36). This partitioning reduces interspecies competition for carbon sources (38).

### 2.4 Social collapse and disease pathogenesis

Microbial social collapse, manifested as dysbiosis—disruption of communal cohesion, functional synergy, and hierarchical equilibrium—directly drives the pathogenesis of multiple human diseases. This collapse is not merely a numerical imbalance of taxa but a breakdown of interspecies cooperation, with dominant “opportunistic” taxa overriding communal metabolic order and triggering host pathological responses. Inflammatory bowel disease (IBD) and obesity represent paradigmatic examples of how microbial social disorganization translates to clinical pathology.

Crohn’s disease (CD), a chronic granulomatous IBD, is defined by catastrophic microbial social cohesion loss, primarily driven by Proteobacteria overgrowth (39). Unlike the balanced competition in healthy guts, CD patients exhibit a increase in Proteobacteria abundance (dominated by *Escherichia coli* clades, especially adherent-invasive *E. coli*, AIEC) (29). These opportunists outcompete anti-inflammatory Clostridia (e.g., *F. prausnitzii*, *Roseburia intestinalis*) through dual metabolic strategies: nitrate respiration and urea metabolism (40). AIEC expresses high levels of urease, converting host urea into ammonia to fuel amino acid synthesis, while depleting nitrogen sources critical for Clostridia survival (41). Metabolomic analysis confirms fecal amino acids (byproducts of bacterial nitrogen metabolism) correlate with Proteobacteria enrichment and disease activity (29). The loss of Clostridia eliminates key anti-inflammatory pathways: *F. prausnitzii* levels decline in active CD, reducing butyrate production and impairing intestinal barrier integrity (42). AIEC further exacerbates collapse by degrading mucin via the Vat-AIEC protease, creating mucosal “gaps” that disrupt spatial segregation and enable invasive colonization (43).

Obesity-associated dysbiosis reflects a distinct form of microbial social collapse: “tyranny of the majority,” where dominant Firmicutes override communal metabolic equilibrium (44). Obesity patients exhibit a 30–50% reduction in gut microbial α-diversity, with Firmicutes/Bacteroidetes ratios shifting from 1–3:1 (healthy) to 4–6:1 (obese) (45). This imbalance is driven by enrichment of Firmicutes taxa like *Megamonas rupellensis*, which monopolize nutrient metabolism to enhance host energy harvest. A multi-cohort study of 1,005 individuals confirmed that *M. rupellensis*-dominated gut communities correlate with 2.1-fold higher obesity risk, via a unique mechanism: degrading intestinal nositol (a natural inhibitor of fatty acid absorption) through the pwy-7237 metabolic pathway (46). In sterile mice colonized with *M. rupellensis*, high-fat diet-induced weight gain and hepatic lipid deposition increased, due to upregulated intestinal fatty acid transporters (46). This “metabolic tyranny” is exacerbated by loss of cooperative taxa: *A. muciniphila* and *Bifidobacterium* decline, eliminating checks on Firmicutes expansion (47). The resultant low-grade inflammation—driven by Firmicutes-derived LPS—further impairs insulin sensitivity, creating a positive feedback loop of microbial disorganization and metabolic dysfunction (48).

### 2.5 The gut-brain axis: collective microbial decision-making

Emerging evidence implicates microbiota social dynamics—rather than individual microbial taxa—in neurobehavioral regulation, with microbial “group intelligence” operating through coordinated metabolic networks and signaling cascades to modulate the gut-brain axis. This collective decision-making is not random but an evolutionarily optimized strategy, where interspecies cooperation generates synergistic signals that shape host cognition, emotion, and stress responses—marking a paradigm shift in understanding neuropsychiatric disorder pathogenesis.

SCFAs, the canonical products of microbial cross-feeding, represent a core mediator of this collective influence (49). Acetate, propionate, and butyrate—synthesized through sequential fermentation of dietary fiber by consortia including *Ruminococcus bromii*, *Bacteroides ovatus*, and *F. prausnitzii* —exert distinct yet complementary effects on brain function (50). Butyrate, primarily produced by Firmicutes taxa, enhances blood-brain barrier (BBB) integrity by upregulating tight junction proteins via inhibiting histone deacetylase 6, reducing BBB permeability in mouse models (51). Propionate, in contrast, acts directly on hypothalamic neurons via the FFAR3 receptor to modulate serotonin synthesis—human gut consortia producing propionate correlate with higher plasma serotonin levels and lower anxiety scores (52). Critically, these effects depend on social cohesion: disrupted cross-feeding in dysbiotic communities reduces total SCFA production by 50–70% and eliminates the propionate-butyrate balance required for normal neuroregulation (53).

Microbial collective action further governs neurotransmitter biosynthesis, the foundation of emotional regulation. The gut microbiota controls 95% of peripheral serotonin production (54): *Clostridium* sp*orogenes* and *Eubacterium rectale* synergistically activate the tryptophan hydroxylase pathway in enterochromaffin cells, generating 5-hydroxytryptophan that crosses the BBB to fuel brain serotonin synthesis (52). Meanwhile, *Lactobacillus* and *Bifidobacterium* strains produce γ-aminobutyric acid (GABA) via glutamate decarboxylase (GAD), with GABA levels in colon contents increase—concentrations sufficient to activate vagus nerve GABA receptors and reduce anxiety (55). This division of labor is tightly regulated: propionate from *F. prausnitzii* upregulates GAD expression in lactobacilli, demonstrating interspecies signaling that coordinates neurotransmitter output (56).

Sterile mouse models provide definitive evidence that structured microbial social networks are requisite for normal neurobehavior (57). Germ-free mice exhibit lower colon serotonin levels, impaired hippocampal neuroplasticity, and heightened anxiety-like behaviors—spending less time in open arms of the elevated plus maze compared to conventional mice (58). Reconstitution with a socially structured consortium (including *Akkermansia muciniphila*, *F. prausnitzii*, and *Bifidobacterium longum*) restores these deficits, with brain-derived neurotrophic factor levels recovering and microRNA profiles in the amygdala normalized (56). In contrast, reconstitution with a disordered community (randomly mixed taxa lacking cross-feeding capacity) fails to reverse anxiety or neurotransmitter imbalances (53), confirming that collective organization—not just microbial presence—drives neuroregulation.

This collective microbial influence extends to neuropsychiatric disorders. Major depressive disorder (MDD) patients exhibit disrupted SCFA cross-feeding networks, with *Prevotella copri* overgrowth suppressing *F. prausnitzii* abundance and reducing propionate production (56). Similarly, autism spectrum disorder (ASD) is linked to impaired microbial tryptophan metabolism: ASD children show lower fecal indole-3-propionate and reduced activation of the brain aromatic hydrocarbon receptor pathway, which regulates synaptic pruning (59). Critically, fecal microbiota transplantation (FMT) with healthy, structured consortia improves MDD scores and ASD social deficits, whereas single-strain probiotics yield minimal benefits—validating the therapeutic potential of targeting microbial social dynamics (60, 61).

## 3 Elucidating the existence of social structures in gut microbial communities

To rigorously validate this hypothesis, a multi-modal experimental and computational framework is essential. First, spatial multi-omics mapping could resolve microbial social networks *in situ* by integrating metatranscriptomics—to identify functionally active taxa and their metabolic interactions—with imaging mass spectrometry, which spatially localizes metabolites and signaling molecules across gut niches. Wang et al. used Slide-seq and MALDI-IMS to identify colonic niches (e.g., Bacteroides-dominant outer mucus, Clostridia-rich crypts) with coordinated quorum sensing, where AI-2 correlated with Bacteroides polysaccharide metabolism (62). Bile acid gradients further structure these neighborhoods, with bile-tolerant Enterobacteriaceae in lumina and sensitive Lactobacillus in crypts (63). This approach would reveal how microbial “neighborhoods” coordinate behaviors, such as quorum-sensing-mediated cooperation or territorial competition, in structurally complex environments like the intestinal crypts or mucosal layers. Second, synthetic microbial societies engineered with defined social hierarchies (e.g., keystone species, subdominant mutualists) could be introduced into gnotobiotic models to study emergent properties, such as community resilience to dietary perturbations or pathogen invasion. For instance, consortia designed to mimic obesity-associated *Firmicutes*-dominant hierarchies could test whether social destabilization drives metabolic dysregulation. Zhang et al. built balanced (keystone Akkermansia, mutualists like Faecalibacterium) and dysbiotic (opportunistic E. coli, reduced Faecalibacterium) consortia. The balanced group restored gut barrier function with higher occludin and glucose homeostasis, while the dysbiotic induced inflammation with higher TNF-α, despite equal richness (64). Third, agent-based dynamic modeling would simulate how competition for resources, cross-feeding dependencies, and environmental stressors (e.g., antibiotics, pH shifts) shape community stability over time. By parameterizing models with empirical data—such as metabolite diffusion rates or interspecies interaction strengths—these simulations could predict tipping points leading to social collapse (e.g., dysbiosis) or identify interventions to restore equilibrium. Li et al. parameterized with empirical data (e.g., B. thetaiotaomicron polysaccharide degradation rate), simulated antibiotic-induced dysbiosis (60% Faecalibacterium decline) and prebiotic rescue (arabinoxylan restored 80% butyrate). And mouse/human validation confirmed these predictions (65). Together, these strategies bridge reductionist experimentation with systems-level analysis, offering a holistic toolkit to decode the rules governing microbial social architectures and their impact on host physiology.

## 4 Implications and future directions

Recognizing the gut microbiota as a socially organized entity revolutionizes our approach to microbiome-targeted therapies. By targeting microbial “governance” mechanisms, we can move beyond blunt interventions like broad-spectrum antibiotics, which indiscriminately disrupt communities, toward precision strategies that restore social equilibrium. For instance, quorum-sensing inhibitors could dismantle pathogenic alliances by blocking bacterial communication—a tactic already showing promise in *Pseudomonas aeruginosa* biofilms, where such inhibitors reduce virulence and biofilm resilience without eradicating commensals (66). Similarly, prebiotics tailored to reinforce cooperative networks might selectively nourish keystone mutualists like *F. prausnitzii*, whose cross-feeding interactions with *A. muciniphila* enhance barrier function and suppress inflammation (67). A study demonstrated that arabinoxylan-oligosaccharides increased butyrate production in dysbiotic patients, correlating with improved metabolic markers—a testament to the power of nurturing microbial teamwork (68). FMT exemplifies the importance of social restoration. While taxonomic diversity is often emphasized, FMT success in treating *Clostridioides difficile* infection hinges on reestablishing functional networks (69). A study found that FMT responders exhibited reconstituted metabolic handoffs, whereas non-responders retained fragmented interaction patterns despite similar diversity (70). This underscores that microbial “societal repair,” not mere species reintroduction, drives therapeutic efficacy. Future research should prioritize socially informed engineering of microbial consortia. Synthetic biology tools could design “diplomat” bacteria engineered to secrete peacekeeper metabolites (e.g., anti-inflammatory molecules or conflict-resolving signals) to stabilize dysbiotic communities. For example, *Lactobacillus reuteri* engineered to produce histamine has been shown to suppress TNF-α in colitis models by modulating host immune-microbe dialogues (71). Additionally, AI-driven social network analysis could map keystone interactions in patient-specific microbiomes, guiding personalized pre/probiotic cocktails.

However, challenges remain. Interventions must avoid unintended consequences—quorum-sensing inhibitors might destabilize beneficial alliances, while engineered strains could face ecological resistance. Longitudinal studies tracking social dynamics during interventions, paired with advanced imaging (e.g., Raman-based *in vivo* metabolic tracking), will be critical to evaluate safety and efficacy. Ethically, as we gain power to manipulate microbial societies, frameworks must ensure these technologies prioritize ecological resilience over forced colonization. Ultimately, viewing the microbiome through a sociological lens bridges ecology, medicine, and systems biology. By decoding the “rules of engagement” within microbial societies, we open pathways to therapies that harmonize, rather than conquer, the invisible civilizations within us.

## 5 Conclusion

The social architecture of gut microbiota constitutes a fundamental biological paradigm that transcends traditional disciplinary boundaries, integrating principles from microbiology and social sciences. This framework provides profound insights into the complex organizational dynamics governing microbial communities, including hierarchical dominance, cooperative networks, and niche specialization (**Figure 1**). Deciphering these sophisticated interactions not only advances our understanding of ecosystem stability but also reveals the mechanistic basis of dysbiosis-related pathologies. Moving forward, targeted manipulation of microbial social structures—through precision modulation of quorum sensing, metabolic cross-feeding, or spatial organization—holds transformative potential for clinical interventions. The development of “socially informed” microbiome therapeutics could pioneer novel strategies for treating inflammatory, metabolic, and neoplastic diseases, ultimately ushering in a new era of ecological medicine. Future research should prioritize high-resolution multi-omics approaches coupled with computational modeling to decode the causal relationships between microbial social dynamics and host physiology.

## Data Availability

The original contributions presented in the study are included in the article/supplementary material. Further inquiries can be directed to the corresponding author.
